# A Reference Methylome Database and Analysis Pipeline to Facilitate Integrative and Comparative Epigenomics

**DOI:** 10.1371/journal.pone.0081148

**Published:** 2013-12-06

**Authors:** Qiang Song, Benjamin Decato, Elizabeth E. Hong, Meng Zhou, Fang Fang, Jianghan Qu, Tyler Garvin, Michael Kessler, Jun Zhou, Andrew D. Smith

**Affiliations:** Molecular and Computational Biology, University of Southern California, Los Angeles, California, United States of America; University of Bonn, Institut of Experimental Hematology and Transfusion Medicine, Germany

## Abstract

DNA methylation is implicated in a surprising diversity of regulatory, evolutionary processes and diseases in eukaryotes. The introduction of whole-genome bisulfite sequencing has enabled the study of DNA methylation at a single-base resolution, revealing many new aspects of DNA methylation and highlighting the usefulness of methylome data in understanding a variety of genomic phenomena. As the number of publicly available whole-genome bisulfite sequencing studies reaches into the hundreds, reliable and convenient tools for comparing and analyzing methylomes become increasingly important. We present MethPipe, a pipeline for both low and high-level methylome analysis, and MethBase, an accompanying database of annotated methylomes from the public domain. Together these resources enable researchers to extract interesting features from methylomes and compare them with those identified in public methylomes in our database.

## Introduction

In eukaryotes, DNA methylation refers to the addition of a methyl group to the 5′ location of cytosines and is closely linked to transcriptional regulation [1]. Lack of methylation in genomic regions usually indicates the accessibility of the underlying DNA sequences to transcription factor binding [2], although recent results have suggested a more complex relationship [3]. Investigating DNA methylation patterns across the entire genomes is a key step to understanding its various roles. There are several techniques to profile DNA methylation, the most comprehensive being whole-genome bisulfite sequencing (WGBS; [4]), which has been used to profile methylation levels across entire genomes in a variety of organisms and cell types [5]–[7]. Alternatively, reduced representation bisulfite sequencing (RRBS) preferentially profiles CpG-rich regions and requires less sequencing [8]. These profiling methods provide single-based resolution for the full epigenome unattainable with ChIP-seq and DNase-seq. For example, recent studies have identified subtle boundary changes in hypo-methylated regions around promoters during cell differentiation – discoveries that would not have been possible without single-base resolution profiling [9], [10]. These experiments provide information for numerous regions of dynamic methylation, believed to related with promoters, enhancers, insulators and more broadly regions dense in transcription factor binding sites [11]. Comparative analysis of the methylomes of different cell types and conditions reveals functional epigenetic domains with implications in cell differentiation and disease onset [10], [12], [13].

The analyses and interpretation of bisulfite sequencing data are usually performed in a multi-stage, multi-resolution manner. Extensive efforts have been devoted to mapping reads and estimating methylation levels at individual cytosine sites [14], [15]. Meanwhile, growing interest is shifting towards biologically meaningful higher-level methylation features, such as hypo-methylated regions (HMRs; [3], [9]), large-scale partially methylated domains (PMDs; [7], [13]), and allele-specific methylated regions (AMRs; [16], [17]). Existing methods to identify these are usually project specific, with few general tools available for down-stream analysis tasks.

As the number of publicly available methylomes rises, large-scale comparative analysis of methylation patterns across multiple samples calls for well-curated reference methylome databases. Some databases exist to address the need for accessing bisulfite sequencing datasets. The NCBI Sequence Read Archive (SRA) contains raw sequences of most BS-seq data [18]. The NGSmethDB database [19] and the NCBI Epigenomics Resources [20] provide pre-computed methylation level results at individual cytosines, saving both time and computational resources to download and analyze those raw reads,. However there are not sufficient effort to address the issue of higher-level methylation features. This creates a computational barrier for researchers who seek to use methylation-based features in large projects. Additionally, databases like SRA and NCBI Epigenetic Resources are designed to accommodate datasets generated from a variety of techniques, including ChIP-seq, RNA-seq and WGBS. As such, their annotation lacks metadata (e.g. bisulfite conversion rates) that are specific to bisulfite sequencing.

MethBase addresses the need for a well-annotated database of methylome features and summary statistics (http://smithlab.usc.edu/methbase/). We collected bisulfite sequencing datasets from SRA and other primary sources and analyzed each with the MethPipe pipeline. From there, we integrated detailed meta information into the UCSC Genome Browser. This provides a convenient avenue through which researchers may select methylomes of interest, visualize them in the UCSC Genome Browser, examine more detailed metadata, and download all available information for additional analysis. MethPipe is a standalone pipeline and freely available open source software, and provides for easy and consistent comparison between private data and public methylomes. Here we explain several aspects of these resources. We also explore a set of general observations that emerge naturally from these resources, demonstrating their power and usefulness in comparative analysis, data integration and technical benchmarking of newly produced methylome datasets.

## Results

### Types of Data in the Database

Individual records in MethBase, referred to here simply as methylomes, correspond to biological samples or replicates, as defined in the original publication of the raw data. When datasets are defined as different technical replicates, for example different libraries made from the same sample, they are pooled in MethBase as one entry. For each methylome, the database provides a variety of data, such as methylation levels of individual cytosines, hypo-methylated regions, partially methylated domains, allele-specific methylated regions, and detailed metadata and summary statistics. We describe these types of data below, and reserve more technical discussion of the pipeline used to generate these data in the Methods section.

#### Methylation levels

Although individual methyl groups can either be present or absent on a cytosine, current WGBS data is not from single cells, and we therefore refer to a methylation “level” for each cytosine, interpreted as the fraction of molecules in the underlying cell population that have the methyl mark on the corresponding cytosine. Since bisulfite sequencing gives a readout for either the presence or absence of a methyl group in each read, we get an unbiased estimate for the methylation level from the ratio of methylated reads to all reads covering a given site. The estimated cytosine methylation level, along with the number of sequenced reads supporting that estimate, is computed for each methylome, along with confidence intervals as described by Hodges et al. [21].

#### Hypo-methylated regions

**Figure 1:**
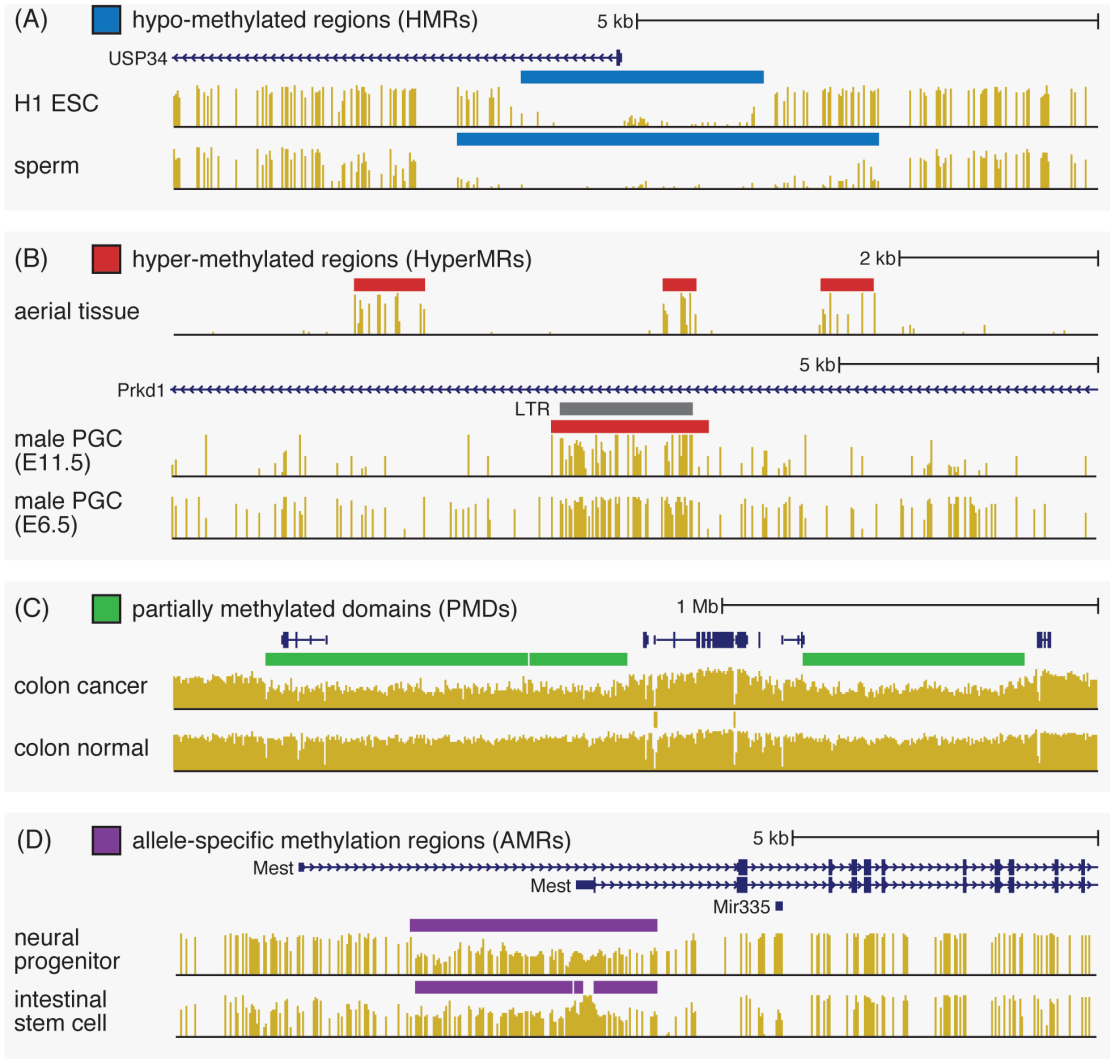
Examples of high-level methylation features available in MethBase through the UCSC Genome Browser track hub. (A) hypo-methylated regions (HMRs); (B) hyper-methylated regions (HyperMRs); (C) partially methylated domains (PMDs), and (D) allele-specific methylated regions (AMRs).

Excluding the brief developmental stages of somatic and germ cell reprogramming [22], mammalian cells typically have high levels of methylation throughout the genome. The more interesting features of mammalian methylomes tend to be those regions lacking methylation (*e.g.* Figure 1A). These hypo-methylated regions (HMRs) have been associated with CpG islands, promoter regions, and more generally enhancers and insulators [11]. We developed a highly accurate method for identifying HMRs using a stochastic segmentation model that accounts for both changes in methylation levels and variance in read coverage along the genome [9]. An individual HMR could represent an interval of the genome that has been protected from methylation, or has been opened up by the activity of specific DNA binding proteins [3]. Regardless of the causes, these intervals are strongly associated with regulation of gene expression, and fluctuations in their boundaries between methylomes likely indicate context-specific regulatory sites [10]. In healthy somatic cell methylomes, the organization of methylation features exhibit strikingly precise boundaries. The precision with which HMRs can be identified is what separates this kind of epigenetic information from others, such as H3K4me marks or DNase hypersensitivity sites, that usually lack precision in boundaries.

#### Hyper-methylated regions

In contrast to mammals, the Arabidopsis genome is devoid of methylation by default, with increased methylation levels localized to specific regions, such as intragenic regions and retrotranposons [23]. A similar pattern, referred to as “mosaic methylation” [24], has been documented in invertebrates. During early development of mammalian germ cells, most of the genome is unmethylated, with certain regions retaining methylation despite global epigenetic reprogramming [25]. In each of these cases, the key features of interest are hyper-methylated regions (HyperMR: *e.g.* Figure 1B), rather than hypo-methylated regions (HMR). Comparative analysis of multiple Arabidopsis methylomes suggests that Arabidopsis HyperMRs, especially those located in intragenic regions, have specific locations that are consistently unmethylated across different ecoytpes and cell types (unpublished studies). These HyperMRs change between different samples in a discrete manner, suggesting that discrete HyperMRs represent a fundamental regulatory and/or functional unit of methylation in plants.

#### Partially methylated domains

One important discovery from early genome-wide investigation of the methylomes of immortalized cell lines (*e.g.* IMR90) was the presence of large partially methylated domains (PMD), that spans hundreds of kilobases [7]. Hansen et al. [12] and Berman et al. [13] also observed such PMDs characteristic of cancer cell lines and primary cancers (*e.g.* Figure 1C), and PMDs has recently been reported in placenta methylomes as well [26]. PMDs have largely conserved locations across samples, and overlap with nuclear lamina associated domains (LAD) and late replicating regions [13], suggesting their involvement in topological organization of chromosomes. To characterize PMDs we employ a hierarchical method that locates PMDs at low resolution followed by further refinement of their boundaries with higher resolution.

#### Allele-specific methylation

In diploid organisms, the two alleles may have different methylation levels in certain regions (*e.g.* Figure 1D). Those allele-specific methylated regions (AMRs) can be either parent-of-origin dependent, or associated with allele-specific sequence variation [16]. The former type of allele-specific methylation is related to gene imprinting [27]. We recently developed a novel computational method to identify AMRs using WGBS data without using genotype to separate reads from different alleles [17]. This method allows a comprehensive investigation of allele-specific methylation on a genome-wide scale. By applying this method to public methylomes in MethBase, we have enabled the study of tissue-specific and organism-specific allele-specific methylation.

### Visualizing and Obtaining Methylation Data from MethBase

**Figure 2:**
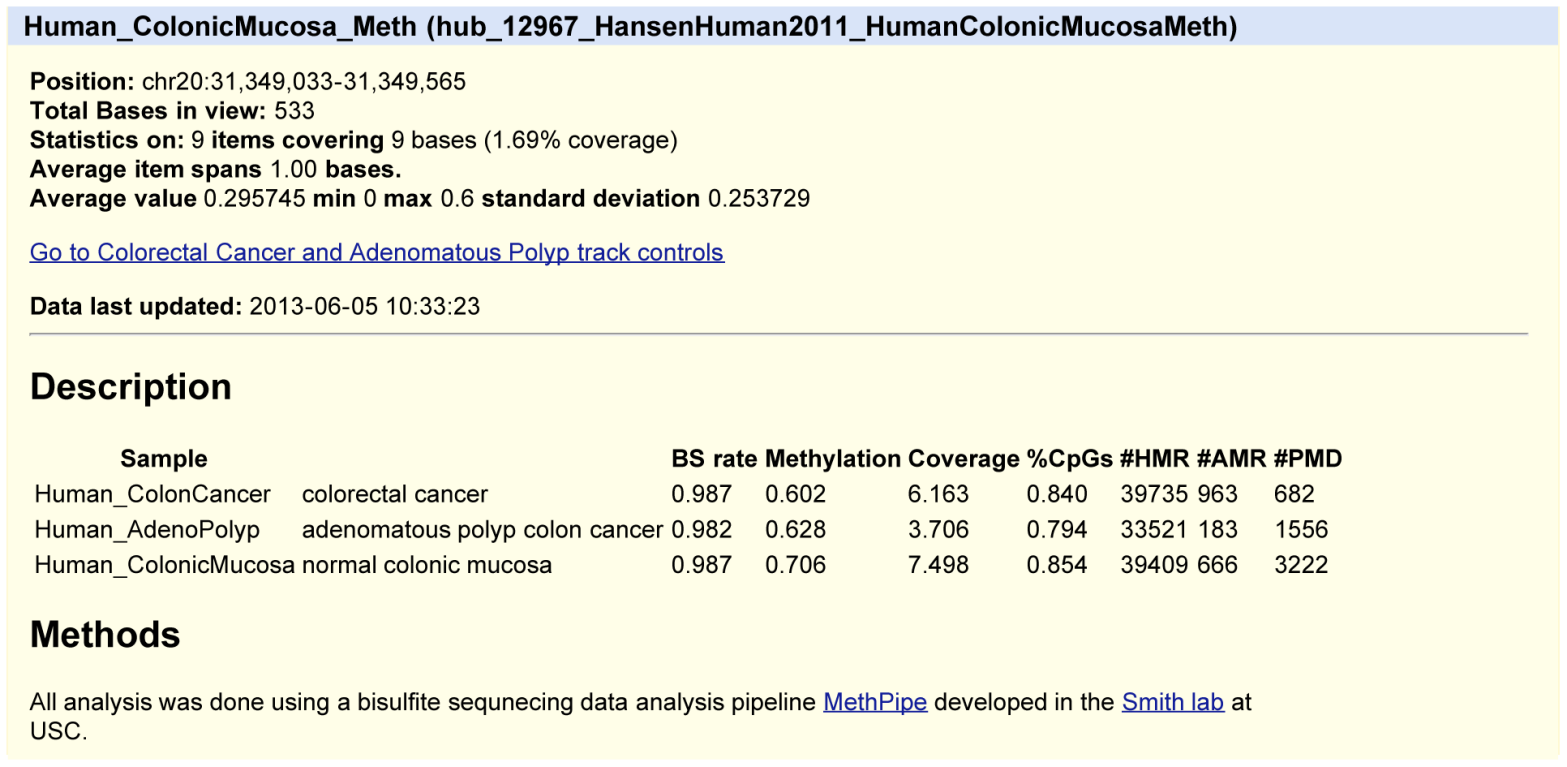
Example of detail meta data and summary statistics of methylation features.

MethBase currently includes over one hundred methylomes from well-studied organisms, including human, mouse, chimpanzee and arabidopsis (studies: 28, 17, 3 and 8; methylomes: 169, 71, 5 and 32, respectively). These methylomes are organized by species and further grouped by the project or publication associated with the data. The processed data in MethBase can be accessed through a Track Hub that can be easily loaded into any mirror of the UCSC Genome Browser [28]. Visual examination of the methylation patterns at specific genes and genomic loci is an essential part of exploratory data analysis for investigators using WGBS data. Pre-computed high-level methylation features, including HMRs, HyperMRs, PMDs and AMRs, highlight potentially interesting regions (Figure 1). From the visualization interface, one may access detailed meta data and summary statistics through the track summary pages (Figure 2). For more detailed genome-scale analysis, researchers also have the option to download the methylation data and pre-computed methylation features through the UCSC Table Browser [29], in the form of either genomic interval annotations or single-site methylation levels.

### Applications

#### Visual exploration of methylation patterns at specific genes and loci

**Figure 3:**
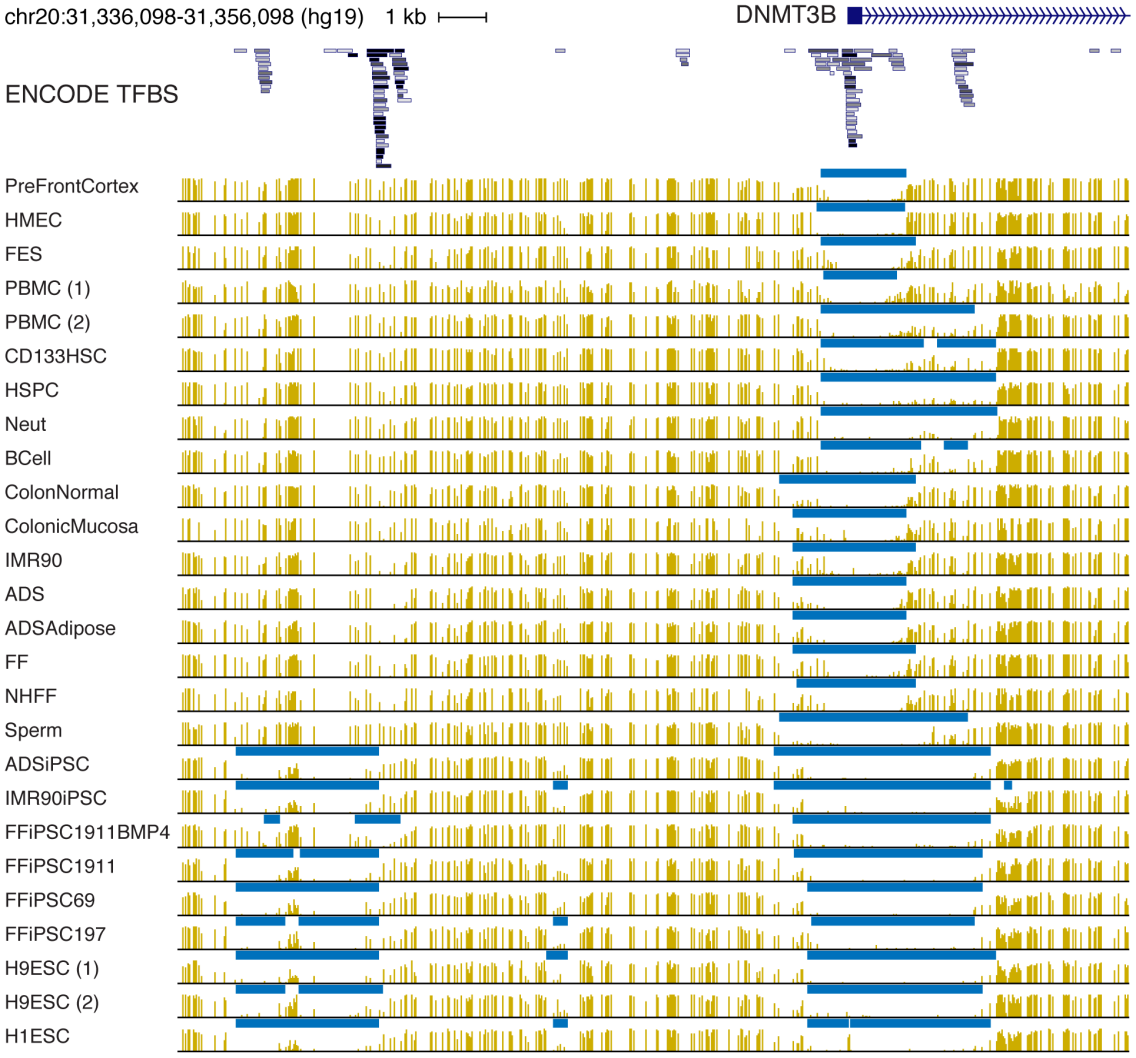
Visualization of methylation profiles and HMRs from MethBase near example gene DNMT3B.

Visual examination of methylation patterns for specific genes and genomic regions is a valuable research tool, and can be applied to differentially expressed genes, evolutionarily conserved elements, or other regions of interest. Using our tools, users may also identify higher-level methylation features from their own methylation data, and compare them with public methylomes in MethBase. Figure 3 shows a genome browser view of the methylation levels and identified HMRs for a subset of human WGBS methylomes in MethBase (disease or mutant samples excluded). The 20 kb genomic interval covers the 5′ end of the DNMT3B gene, a *de novo* methyltransferase whose absense in mice is embryonic lethal due to developmental defects [30]. Displayed below the partial DNMT3B gene model are transcription factor binding sites (TFBS) identified by the ENCODE project [31]. Among the 26 methylomes displayed below, 9 are from ESCs and iPSCs, and 6 from blood cells, with the rest of diverse cellular origin. In each methylome, an HMR overlaps the transcription start site (TSS) of DNMT3B, but the size of these HMRs and the positioning of their boundaries exhibit interesting variation. In the ESC and iPSC methylomes, as well as 5 of the blood methylomes, this HMR extends downstream of the DNMT3B TSS by roughly 3 kb. In the remaining methylomes the downstream boundary is roughly 1.2 kb downstream of the TSS. The extended portion of this HMR clearly overlaps a cluster of TFBS, suggesting a regulatory module functioning in iPSC, ESC and blood cells. At the other end of this promoter HMR the boundaries seem to form discrete categories, suggesting several distinct regulatory modules with precise boundaries. Another HMR appears roughly 10 kb upstream of the DNMT3B TSS exclusively in the iPSC and ESC methylomes. Such these observational features in conjunction with other forms of genome-wide data provide a starting point for dissecting the architecture of particular regulatory regions.

#### Comparative analysis of HMRs

**Figure 4:**
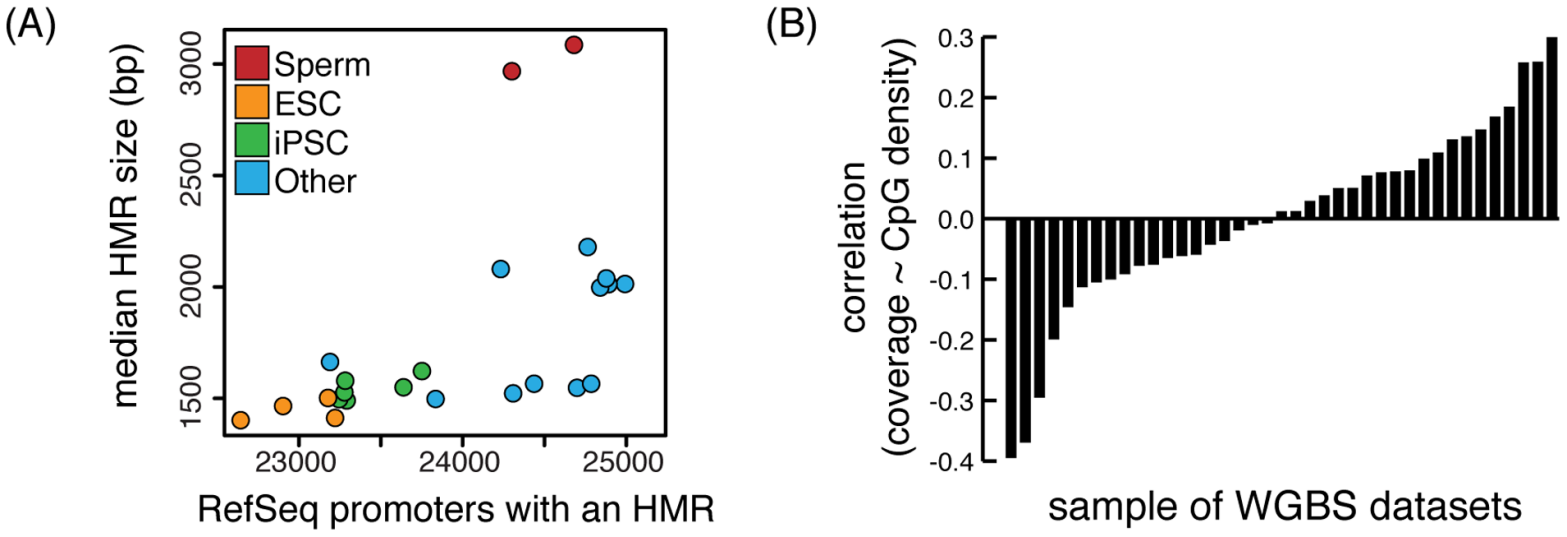
Comparing biological and technical features of methylome data. (A) Human methylomes are clustered according to the number and size of promoter HMRs. (B) Correlation between depth of coverage at CpG sites and CpG densities in 1 kb windows for a subset of human methylomes from MethBase. X-axis indicates the index of methylomes sorted by their correlation coefficients. See Table S1 for *p*-values and the list of samples.

Pre-computed methylation features can be directly downloaded from MethBase, most in the form of genomic intervals, permitting further analyses and/or integration with other data. For example, Hodges et al. [10] studied the differences of HMRs between sperm, embryonic stem cells and blood cells. This is easily extended to many more cell types using MethBase. The number and size of HMRs provide a concise high-level summary of each methylome. Based on the number and size of promoter HMRs, methylomes of embryonic stem cells (ESC) cluster together, and the methylomes of induced pluripotent stem cells (iPSC) form another cluster, with the methylomes of differentiated cells showing greater variance (Figure 4A). Interestingly, iPSC methylomes have a slightly larger number of promoter HMRs than those of ESCs, perhaps suggesting incomplete induction of pluripotency. Consistent with the observation by Molaro et al. [9], promoter HMRs in sperm are much wider and more numerous than in other cell types.

#### Empirical benchmarks for new WGBS experiments

Technical characteristics of publicly available WGBS methylomes vary considerably. Part of this results from the decisions made by investigators concerning parameters such as read length and sequencing depth. Additional variation comes from poor experimental outcomes, reflected in low bisulfite conversion, or read coverage that is biased towards or away from CpG-dense regions. This variation raises the question of how to evaluate the quality of methylomes from WGBS or RRBS experiments. The large and growing collection of methylomes in MethBase provides an empirical benchmark for future investigations. One potentially important feature that varies perhaps unexpectedly is the relationship between CpG density and coverage depth in WGBS data. Using the methylation data from a subset of human WGBS methylomes, we computed the average coverage at CpG sites and the CpG density in each 1 kb bin through the human autosomes, which surprisingly shows significant correlation in a large number of samples (Figure 4B and Table S1). The correlation may be either positive or negative, even for samples from the same study (Table S1). It remains unclear whether such correlation reflects technical artifacts or any underlying biological phenomena. However, methylation features have several well-known relationships with CpG density, and our confidence in methylation level estimates is a function of depth of coverage. The unexpected correlation between read coverage and CpG density may affect our ability to assess the methylation status of regions with varying CpG density.

#### Using evolutionary information to study observed methylation phenomena

**Figure 5:**
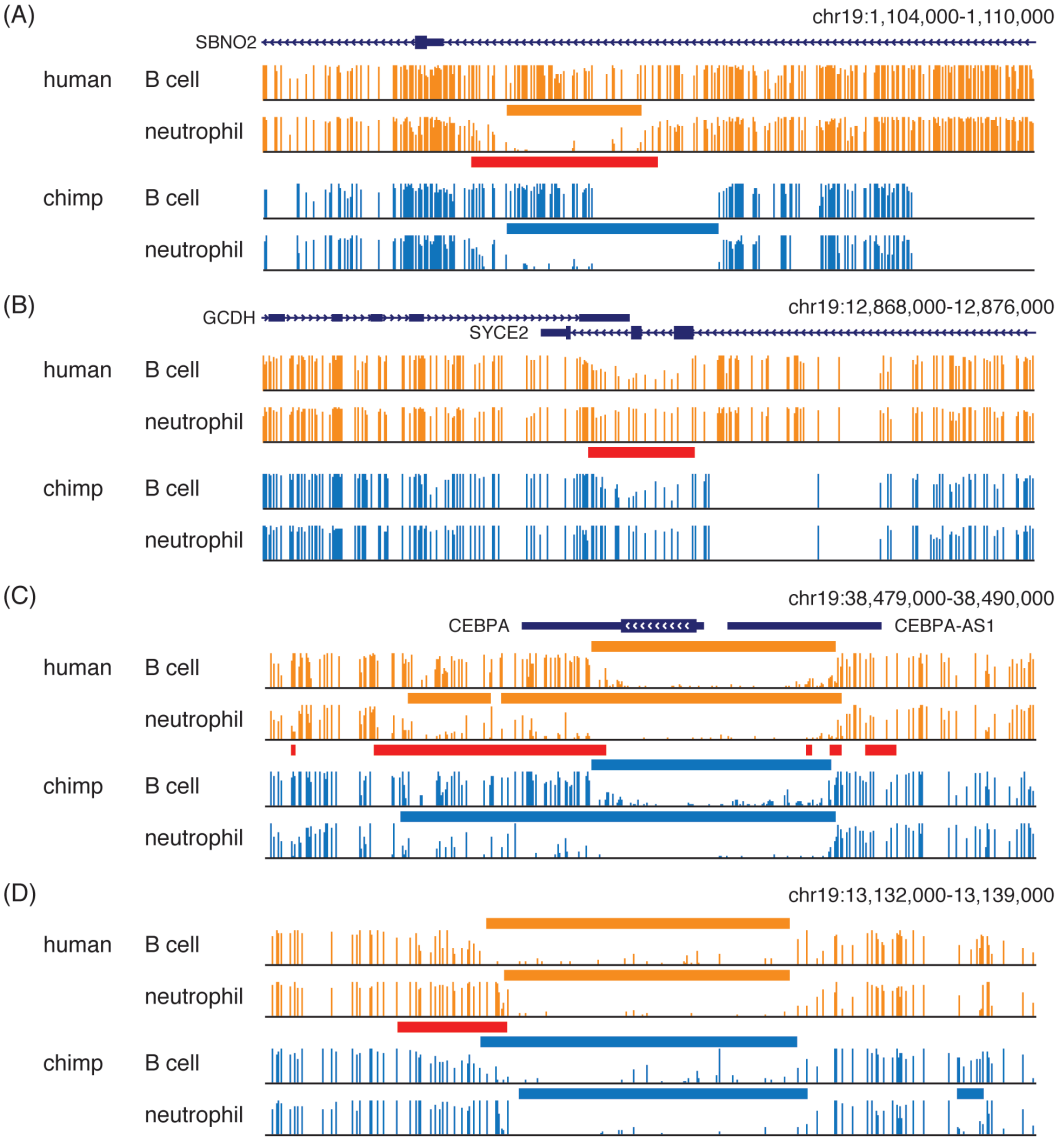
Evolutionary support for commonly observed types of differential methylation. (A) differential presense of an HMR; (B) partial difference in methylation level; (C) shifting of HMR boundaries; (D) difference in precision of HMR boundaries. Orange bars: HMRs in human; Blue bars: HMRs in chimp; Red bars: DMRs between B cells and neutrophils.

DNA methylation patterns are conserved to varying degrees between species [9], [10], and methylomes in MethBase are mapped between species to facilitate evolutionary analysis. For example, we compared B cell and neutrophil methylomes, both of which are available in the two species [10], and the methylation states of chimpanzee are mapped to orthologous sites in the human genome (see Methods). Applying tools from MethPipe, we identified differentially methylated regions between B cells and neutrophils within human and within chimpanzee. These differentially methylated regions provide evolutionary support for several of the typical scenarios of methylation dynamics (Figure 5). Figure 5A shows the situation of an existing HMR disappearing in one methylome, or a new HMR emerging in the other. Figure 5B illustrates a partial change in methylation level through a genomic interval. In Figure 5C, the boundaries of an existing HMR shift, resulting in the expansion of an HMR in one methylome (or shrinkage in the other methylome). It is also possible that the location of boundaries of a HMR remains the same, but changes from a sharp transition to a gradual transition (Figure 5D). The partial methylation reduction and the boundary property change are subtle methylation changes. As with strong sequence conservation, these conserved differential methylation patterns between human and chimpanzee suggest that these regions have context-specific functions.

## Discussion

MethBase and MethPipe are powerful resources for projects seeking to characterize additional methylomes or leveraging existing methylomes in genome-scale studies. MethBase allows researchers to easily access pre-computed methylome features and meta-information specific to WGBS experiments. As the number of methylomes analyzed grows, MethBase facilitates comparative analysis of methylation patterns across cell types, species and disease conditions. When new methylomes are produced, these resources also help define quality control benchmarks. By viewing MethBase data through the UCSC Genome Browser, subtle and context-specific changes can be identified in a targeted manner.

The MethPipe pipeline used to create the database is available as a stand-alone open source software package. This allows researchers to extend their analysis of high-level methylation features such as HMRs, HyperMRs, PMDs and AMRs to their own studies involving new methylomes. Importantly, the features identified in new unpublished methylomes can be directly compared with all previously published data sets in MethBase without the need to fully re-analyze each. The pipeline is modular, containing tools both for basic processing of bisulfite sequencing reads, and for identifying and comparing higher level methylation features. This allows users to choose which analyses to perform and to easily integrate their own. The MethPipe tools have been used in several studies [32]–[34], and are now extensively validated and documented.

Our database complements other tools and databases with its addition of high-level methylation features. By identifying these features directly from bisulfite sequencing data without reference to annotated genomic features, we enable unbiased assessment of the methylation landscape. In addition, these methylation features can be investigated accurately at single-base resolution. MethPipe currently calls HMRs, HyperMRs, PMDs and AMRs, but there are certainly other interesting methylation features that remain to incorporated. For example, Xie et al. recently reported a distinct class of genomic regions, called DNA methylation valleys, which contain early developmental regulatory genes [35]. Depending on the cell types and resolution, novel categories of higher-level methylation features may be characterized, and will likely lead to the development of new analytic methods.

## Methods

We created the MethBase database using raw sequenced reads downloaded from the NCBI SRA and other public repositories of WGBS datasets. The raw sequence data are processed through the MethPipe pipeline (http://smithlab.usc.edu/methpipe/), which includes both published algorithms and newly developed methods. Here we describe the methodology and rationale for each major step in the analysis process.

### Mapping Bisulfite Sequencing Reads

Bisulfite treatment converts unmethylated cytosines in the original DNA fragments into uracils, and as a result thymines in treated, sequenced reads originate from either thymines or unmethylated cytosines. There are two approaches for mapping WGBS reads: the three-alphabet approach and the wild card approach [14]. We used the rmapbs program, one of the wildcard based mappers [36]. Input reads are filtered by their quality, and adapter sequences in the 3′ end of reads are trimmed before mapping. Next, uniquely mapped reads with mismatches below a certain threshold are kept for further analysis. For pair-end reads, two mates from the same original DNA segment are mapped simultaneously. The rmapbs-pe program checks the appropriate configuration of the mate pairs. If the two mates overlap, the overlapping part of the mate with lower quality is clipped. This procedure prevents double counting. Users may also use alternative mappers (see [14] and [15] for reviews). Our pipeline provides a program that conveniently converts the output of alternative mappers to the format supported by MethPipe.

### Removing Duplicate Reads

Duplicate reads are generated from a single original DNA fragment, and usually present in large quantities, possibly due to preferential amplification by PCR (Cokus et al. called them “clonal reads” [6]). If we keep duplicate reads, especially when those over-amplified DNA fragments have different methylation state from other DNA molecules, the estimation of methylation levels is biased toward the over-amplified DNA molecules. Duplicate reads always map exactly to the same genomic location (same chromosome, same starting position, same ending position and same strand). We use the duplicate-remover program to randomly select one from multiple duplicate reads as the representative of the original DNA fragment. This correction is conservative, because reads mapping to the same location may actually represent distinct DNA fragments, and unbiased, because those retained reads are chosen randomly. The duplicate-removal procedure is done on a per-library basis, as any reads from different libraries are necessarily from distinct molecules. We pool all reads from multiple sequencing runs of the same biological library before removing duplicates as described above.

### Estimating Methylation Levels

After reads are mapped and filtered, the methcounts program is used to obtain read coverage and estimate methylation levels at individual cytosine sites. We count the number of methylated reads (containing C’s) and the number of unmethylated reads (containing T’s) at each cytosine site. The methylation level of that cytosine is estimated with the ratio of methylated to total reads covering that cytosine. For cytosines within the symmetric CpG sequence context, reads from the both strands are used to give a single estimate. Besides methylation levels at individual cytosines, researchers are also interested in the methylation status in certain regions, which can be represented with mean methylation level, proportion of methylated cytosines or mean methylation level weighted by coverage [37]. These values can be computed for any single region using the levels program, and the weighted mean methylation level can be computed for a set of regions (such as promoters) using roimethstat.

Based on methylation levels and coverage, MethBase also includes several useful and easily computed summary statistics for each sample: mean coverage, genome-wide mean methylation level, number of covered CpG sites and number of covered cytosines in the reference genome.

### Estimating Bisulfite Conversion Rate

Sodium bisulfite converts unmethylated cytosines in DNA molecules to uracils, which are read out as thymines during sequencing. However depending on the treatment time and/or experimental conditions, the conversion may not be complete, leaving certain unmethylated cytosines as C’s. The bisulfite conversion rate, defined as the rate at which unmethylated cytosines in the sample appear as T’s in the sequenced reads, is an important measure of the quality of a WGBS experiment. Estimating bisulfite conversion rate requires a priori knowledge of the methylation status on at least a portion of the cytosines in the sample. One typical technique is to spike in some DNA that is known to be unmethylated, such as a Lambda virus, when preparing sequencing libraries. Alternatively, one may use other unmethylated cytosines, such as the those in chloroplast DNA of plants or mitochondrial DNA of humans [32]. We count the number of converted reads (containing T’s) and the total number of reads covering those unmethylated cytosines. The ratio of converted reads to all reads gives the estimates of the bisulfite conversion rate. The method is implemented in the bsrate program.

### Identifying Hypo-methylated Regions

From the above analysis, we obtain the methylation level and coverage at individual cytosines along the genome, which are used to identify higher-level methylation features, such as HMRs (see above). We employ a two-state HMM-based method to identify HMRs in mammalian methylomes [9]. This model introduces one state representing hypo-methylated regions, and another state representing highly methylated background. Since the estimated methylation levels suffer large variance due to randomly sampled molecules (especially at lower coverage), we directly model the observed read counts with a beta-binomial distribution, for which we implemented a rapid and numerically stable parameter estimation method. The HMM is trained using the Baum-Welch algorithm [38], and posterior decoding identifies HMRs. The method is implemented in the hmr program. Median HMR size and the number of HMRs directly result from this computation, and can provide a quick and useful characterization of a mammalian methylome.

### Identifying Hyper-methylated Regions

HyperMRs are also inferred from the methylation level and coverage at individual cytosines, with a stochastic segmentation model similar to the HMR-finding method. Visual examination of methylation patterns of multiple samples motivated us to model the methylation status of cytosines in the Arabidopsis genome with three states: the *hypo*-methylated state in the background, the *HYPER*-methylated state in HyperMRs and the *HYPO*-methylated state scattered within HyperMRs. In addition, the length of genomic regions is modeled with explicit duration HMM (“variable duration” HMM; [39]). Explicit duration distribution generalizes the implicit geometric duration distribution in HMM, and is more flexible. Detailed mathematical formulation is given in supplementary material. The method is implemented in hmr_plant program.

### Identifying Partially Methylated Domains

As explained above, PMDs are large-scale methylation features found in immortalized cell lines and cancerous cells. We developed a hierarchical method to identify PMDs. First, the genome is divided into 1 kb non-overlapping bins (an empirically determined value that remains user-adjustable). We count the number of methylated and unmethylated observations (*i.e.* CpG states in reads) in each bin. Similar to the HMR-finding method, we use a two-state HMM model to segment the genome into PMDs and background regions. This step at low resolution gives the location of PMDs, while the exact location of their boundaries need further refinement. Since the boundary between a PMD and a non-PMD region must reside within the two bins at the end of that PMD and that non-PMD region, the refinement of PMD boundaries reduces to detecting a single change-point along the sequence of sites in those two bins. The single change-point detection problem has been extensively studied, and we used the binary segmentation procedure of Sen et al. [40].

### Identifying Allele-specific Methylated Regions

To identify allele-specific methylated regions, we use the linkage information of the methylation status between adjacent cytosines in a read. The separation of reads into two alleles and the testing of whether a certain region fits the allele-specific model is carried out with the statistical method described by Fang et al. [17]. Additionally, a single-site profile for an allele-specific methylation “score” can be computed along the genome by testing for significance of linkage between methylation status in reads covering adjacent CpGs. The programs for identifying AMRs and computing allelic scores are amrfinder and allelicmeth.

### Identifying Differentially Methylated Regions

Comparative studies of methylomes usually involves identifying DMRs between samples from different conditions. MethPipe includes two different methods to identify DMRs between two methylomes, each with its applicable situations. The first method extends that introduced by [10]. Differential methylation scores are first computed at individual CpG sites, taking into account observed frequencies of methylation as well as the amount of data contributing to those frequencies [41]. Next, non-overlapping parts of HMRs (*i.e.* the contiguous parts of the symmetric difference of the two interval sets) are evaluated to ensure they contain a sufficient number of differentially methylated CpG sites. This strategy is HMR-centric, and makes sure that the absense of an HMR in one methylome is not simply due to lack of data. This is implemented in the program dmr. The second method uses a three-state HMM to segment the methylome into contiguous sets of CpGs that are either (i) not differentially methylated, (ii) have an over-abundance of significantly different CpG sites in one direction, and (iii) have an over-abundance in the other direction (details in supplementary material). The second method is sensitive to DMRs caused by partial methylation variation, and is implemented in the dmr2 program.

### Cross-species Comparison of Methylomes

To facilitate cross-species comparison of methylomes of multiple species, we converted the methylation data of mouse and chimpanzee to the corresponding locations in the human genome. The liftOver tool provided by UCSC Genome Browser http://genome.ucsc.edu/cgi-bin/hgLiftOver is used to directly convert the methylation level file at individual cytosines (output from the program methcounts ) to the human genome. Next we rerun the hmr and pmd programs on the converted methylation data file. Since the program amr works on the mapped read file, we directly liftover the list of AMRs with the liftOver program.

## Supporting Information

Figure S1 **The effect of coverage on HMR identification measured using Jaccard’s index.**
(TIF)

Table S1 **Correlation between depth of coverage and CpG densities.**
(PDF)

File S1 **Supplementary information including supplementary methods and results.**
(PDF)
