# Supplementary figures and images for: A Reference Methylome Database and Analysis Pipeline to Facilitate Integrative and Comparative Epigenomics
Source: PLoS One. 2013 Dec 6;8(12):e81148. doi: 10.1371/journal.pone.0081148 (PMC3855694; doi:10.1371/journal.pone.0081148)

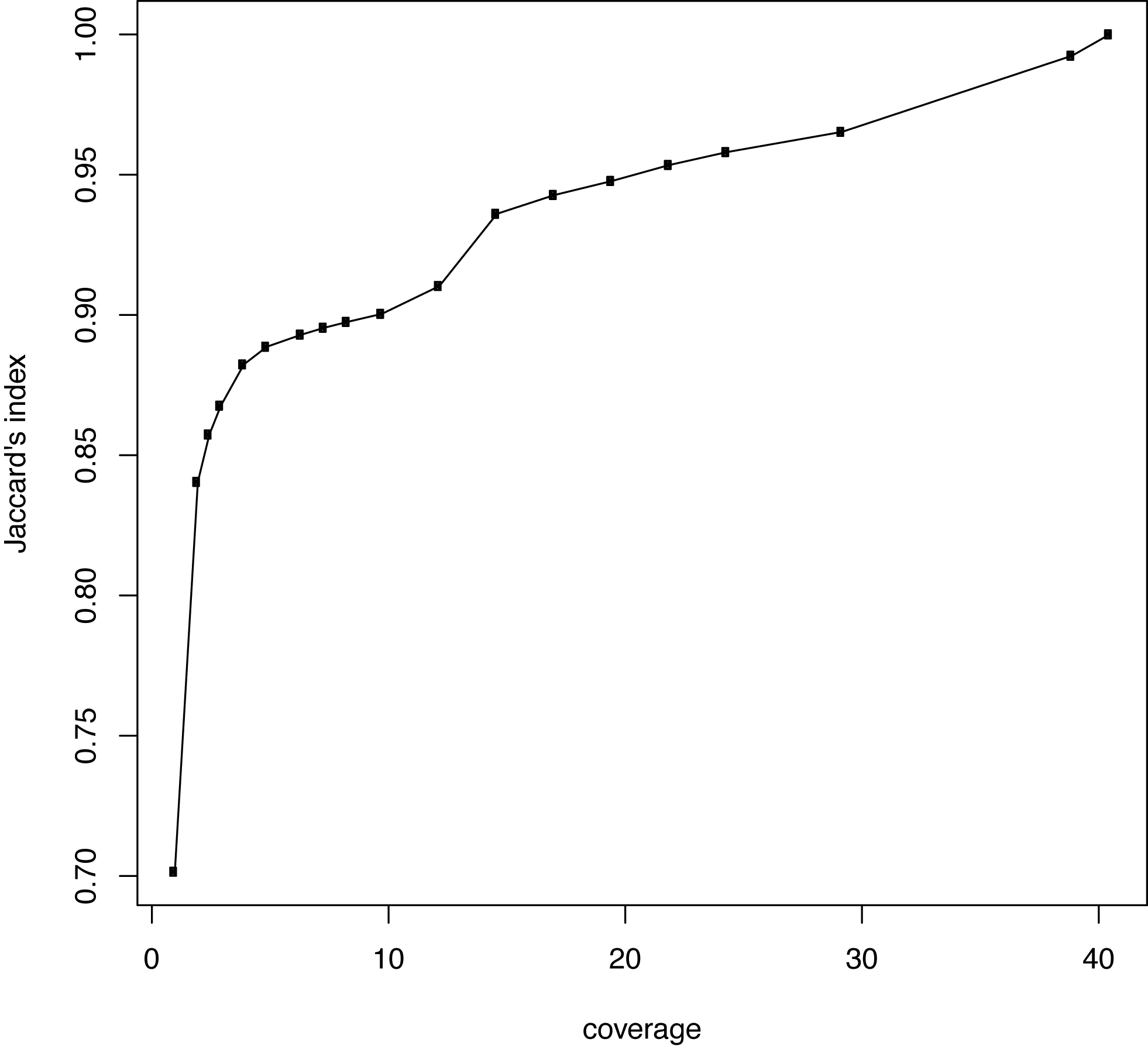

Supplement: Figure S1 — The effect of coverage on HMR identification measured using Jaccard’s index. (TIF) [file pone.0081148.s001.tif]
