# Supplementary material for: A Reference Methylome Database and Analysis Pipeline to Facilitate Integrative and Comparative Epigenomics
Source: PLoS One. 2013 Dec 6;8(12):e81148. doi: 10.1371/journal.pone.0081148 (PMC3855694; doi:10.1371/journal.pone.0081148)
Supplement: Table S1 — Correlation between depth of coverage and CpG densities. (PDF) [file pone.0081148.s002.pdf]

References	Samples	Correlation	p-value
Lister et al. (2009)	H1ESC	-0.3936	0
	IMR90	-0.3683	0
Lister et al. (2011)	ADS	-0.0178	55e-198
	ADSAdipose	-0.0061	1e-24
	ADSiPSC	0.0979	0
	FF	-0.1978	0
	FFiPSC1911	0.0114	0
	FFiPSC1911BMP4	-0.1449	0
	FFiPSC197	-0.0355	0
	FFiPSC69	-0.0418	0
	IMR90iPSC	-0.0745	0
Hon et al. (2012)	HMEC	-0.0636	0
	HCC1954	-0.0089	3.1e-51
Heyn et al. (2012b)	BCell-Healthy	0.0701	0
	BCell-ICF	0.1460	0
Heyn et al. (2012a)	CD4T-100yr	0.0497	0
	CD4T-Newborn	0.0372	0
Hansen et al. (2011)	ColonCancer	-0.0902	0
	ColonicMucosa	-0.0989	0
Berman et al. (2012)	ColonNormal	-0.0762	0
Hodges et al. (2011)	BCell	-0.0603	0
	CD133HSC	0.0108	0
	HSPC	-0.2941	0
	Neut	0.0753	0
Molaro et al. (2011)	Sperm	0.1296	0
Laurent et al. (2010)	FES	0.0766	0
	H9ESC	-0.1120	0
	NHFF	0.0786	0
Martins-Taylor et al. (2012)	NE-DNMT3BKO	0.1835	0
	NE-mis24	0.2577	0
	ESC-DNMT3BKO	0.1673	0
	ESC-mis24	0.2566	0
Li et al. (2010)	PBMC	0.1347	0
Zeng et al. (2012)	PreFrontCortex	-0.0581	0
Schroeder et al. (2011)	SHSY5Y	0.1082	0
	Cortex	0.0281	0

Table S1: Correlation between depth of coverage and CpG densities.
