# Supplementary material for: A Reference Methylome Database and Analysis Pipeline to Facilitate Integrative and Comparative Epigenomics
Source: PLoS One. 2013 Dec 6;8(12):e81148. doi: 10.1371/journal.pone.0081148 (PMC3855694; doi:10.1371/journal.pone.0081148)
Supplement: File S1 — Supplementary information including supplementary methods and results. (PDF) [file pone.0081148.s003.pdf]

Supplementary Material

Qiang Song [1], Benjamin Decato [1], Elizabeth E. Hong[1], Meng Zhou[1], Fang Fang[1,2], Jianghan Qu[1], Tyler Garvin[1], Michael Kessler[1], Jun Zhou[1], Andrew D. Smith[1,*]

1 Molecular and Computational Biology, University of Southern California, Los Angeles, CA 90089 USA

2 Current address: Lineberger Comprehensive Cancer Center, University of North Carolina at Chapel Hill, Chapel Hill, NC 27599 USA

∗ E-mail: andrewds@usc.edu

Supplementary Methods

We describe two new methods for identifying hyper-methylated regions (HyperMR) and differentially methylated regions (DMR) that were not published previously.

S1. Identifying hyper-methylated regions

In plant methylomes and other methylomes showing "mosaic methylation" pattern, localized regions of increased methylation are of interest. The input data are a sequence of read counts at each CpG site ordered by their genomic locations, $\mathcal{X} = \{(m_i, u_i)\}$, where m_i and u_i denote the number of methylated and unmethylated reads at the ith CpG site. Through comparative analysis of multiple Arabidopsis methylomes, we observed that Arabidopsis HyperMRs, especially those located in intragenic regions, have specific locations that are consistently unmethylated across different ecoytpes and cell types (unpublished studies). We therefore model the methylation status of cytosines in the Arabidopsis with three states: the *hypo*-methylated state in the background, the *HYPER*-methylated state in HyperMRs and the *HYPO*-methylated state scattered within HyperMRs. We denote the hidden states with $\mathcal{Q} = \{q_i\}$, where q_i is the hidden state of the ith observation. The locations where the methylation states change is denoted with $\mathcal{C} = \{c_0, \ldots, c_j, \ldots, c_m\}$, where c_0 points to the first observation, and c_m the last observation. We use $b_{q_i}(X_i)$ to denote the likelihood of the ith observation in hidden state q_i (modeled with the beta-binomial distribution), and $d_q(l)$ to denote the likelihood of the length l of a sequence in the hidden state q. The complete likelihood of the input data assuming all model parameters known is

$$p(\mathcal{X}, \mathcal{Q}, \mathcal{C}|\Theta) = \prod_{j=1}^{m} \big(a_{q_{c_{j-2}} q_{c_{j-1}}} d_{q_{c_{j-1}}}(c_j - c_{j-1}) \prod_{i=c_{j-1}}^{c_j - 1} \text{BetaBin}(m_i | m_i + u_i, \alpha_{q_i}, \beta_{q_i})\big). \tag{1}$$

The question is to estimate the model parameters Θ and obtain the posterior estimates of missing data $\mathcal{Q}$ and $\mathcal{C}$ from the observed data $\mathcal{X}$. We train the model with a method similar to the Baum-Welch algorithm [1]. The forward score $\alpha_i(q)$, the likelihood of the observations $X_{1:i}$ with the ith observation being the end of a segment in hidden state q, is

$$\alpha_i(q) = \sum_{j=1}^{i-1} \sum_{s \neq q} \alpha_j(s) a_{sq} d_q(i-j) \prod_{k=j+1}^{i} b_q(X_k).$$

The backward score $\beta_i(q)$, the likelihood of the observation $Y_{i+1:N}$ given the ith observation being the end of a segment in hidden state q, is

$$\beta_i(q) = \sum_{j=i+1}^{N} \sum_{s \neq q} a_{qs} d_s(i-j) \prod_{k=i+1}^{j} \big(b_s(X_k)\beta_j(s)\big).$$

We compute the forward scores and backward scores recursively. The posterior probability of the ith observation being in state q is

$$\gamma_i(q) = \frac{\alpha_i(q)\beta_i(q)}{\sum_{q:\text{all hidden states}} \alpha_i(q)\beta_i(q)}.$$

With the posterior probabilities above, we update the estimates of parameters of the emission distributions as described in [2]. To estimate the parameters of the duration distributions, we perform a posterior segmentation. A cytosine site is assigned to the state of the largest posterior probability. Adjacent cytosines with the same state are joined into a segment. The lengths of segments are computed in terms of the number of cytosine sites, which are used to estimate the parameters of the duration distributions. We iterate the above steps until the change of the complete likelihood (Eq. 1) is below a given threshold. After model training, we divide the genome into HyperMRs and non-HyperMR with either posterior decoding or the Viterbi algorithm [3]. Because of the explicit duration distribution, the time complexity is quadratic in the term of the number of CpG sites. In actual implementation, we impose a cutoff on the maximum length of each region.

S2. Identifying differentially methylated regions

MethPipe provides two programs for identifying differentially methylated regions: the HMR-centric `dmr` algorithm, and the `dmr2` program implementing a three-state VDHMM model to infer the boundary of DMRs. The latter method is independent of pre-defined HMRs, and sensitive to DMRs due to partial methylation variation and changes in boundary properties. The method builds up on the differential methylation scores (diff-score) for individual cytosine sites [4]. Given two samples, we compute the diff-score at each cytosine site, and the sequence of diff-scores (denoted with $\mathcal{Y} = \{y_i\}$) is used as the input data for the following VDHMM model. Since the diff-score is a probability, ranging from 0 to 1, the beta distribution is used to model these observations.

With two methylomes, there are three possible situations at a cytosine site: (i) no differential methylation, (ii) methylome 1 has lower methylation, and (iii) methylome 2 has lower methylation. We therefore introduce three states, namely *same* (s), *gain* (g) and *loss* (ℓ) . For convenience, we use the language of gain and loss of methylation between samples, but remark that in many cases there will is no time dependence. Since a region gaining methylation is unlikely adjacent to a region losing methylation, we forbid the transition between g and l states. The transition matrix for this model is

$$\begin{bmatrix} p_{gg} & 1-p_{gg} & 0 \\ p_{sg} & p_{ss} & p_{s\ell} \\ 0 & 1-p_{\ell\ell} & p_{\ell\ell} \end{bmatrix}.$$

The procedures for model training and segmentation are similar to the three-state VDHMM for identifying HyperMRs, except the emission distribution of the *diff-scores* is the beta distribution. From the three-state VDHMM model, we obtain a set of DMRs. To assess the statistical significance of these DMRs, we assign empirical p-values to them based on a randomization method. For a DMR covering the sth CpG to the tth CpG, we compute the area of methylation difference between those two samples as following:

$$v = \sum_{i=s}^{t} |f_{i1} - f_{i2}|,$$

where f_{i1} and f_{i2} represent the methylation frequencies of the ith CpG in sample 1 and sample 2 respectively. Next we randomly sample r genomic regions with the same number of cytosines, and compute the area of methylation difference for these r regions (often in practice $r = 50,000$). We assign p-values to DMRs based on the empirical distribution from the r random regions, and only DMRs with p-value below a certain threshold are reported

Supplementary results

S3. Effect of coverage

We performed down-sampling analysis to evaluate the effect of coverage on the identification of HMRs. We selected a deep-sequencing sample from [5], and obtained a series of down-sampled datasets with coverage ranging between 1 and 40. The hmr program is used to find HMRs in each down-sampled dataset respectively with the default parameters. The set of HMRs from the dataset with the highest coverage (∼40x) are considered as "golden-standard". We used the Jaccard's index to measure the similarity of the set of HRMs in down-sampled data to the "golden-standard". Specifically, the Jaccard's index is defined as the proportion of common CpG sites covered by both sets of HMRs among those CpG sites covered by any HMRs:

$$J(A, B) = \frac{|A \cap B|}{|A \cup B|},$$

where A denotes the set of CpG sites covered by the "golden-standard" and B the set of CpGs sites covered by the other set of HMRs. As shown in Figure S1, the Jaccard's index is 0.95 at ∼20x, and 0.90 at ∼10x. The performance drops sharply for coverage below ∼5x, but we are still able to achieve the Jaccard's index at 0.72 when the coverage is as low as ∼1x. Our methods for identifying PMDs and HyperMRs are similar, and the above result also holds. The required coverage therefore is even lower since the PMD-finding method internally works by accumulating CpGs in fixed-length bins. The performance of AMR-finding algorithm depends on both coverage and read length, which was addressed in [6].

Supplementary Tables

Table S1 shows the correlation between depth of coverage and CpG densities in human WGBS samples.

References

1. Baum LE, Petrie T, Soules G, Weiss N (1970) A Maximization Technique Occurring in the Statistical Analysis of Probabilistic Functions of Markov Chains. The Annals of Mathematical Statistics 41: 164–171.

2. Molaro A, Hodges E, Fang F, Song Q, McCombie WR, et al. (2011) Sperm methylation profiles reveal features of epigenetic inheritance and evolution in primates. Cell 146: 1029–1041.

3. Viterbi A (1967) Error bounds for convolutional codes and an asymptotically optimum decoding algorithm. IEEE Trans Inf Theor 13: 260–269.

4. Hodges E, Molaro A, Dos Santos CO, Thekkat P, Song Q, et al. (2011) Directional DNA methylation changes and complex intermediate states accompany lineage specificity in the adult hematopoietic compartment. Molecular Cell 44: 17–28.

5. Xie W, Schultz MD, Lister R, Hou Z, Rajagopal N, et al. (2013) Epigenomic analysis of multilineage differentiation of human embryonic stem cells. Cell .

6. Fang F, Hodges E, Molaro A, Dean M, Hannon GJ, et al. (2012) Genomic landscape of human allele-specific DNA methylation. Proceedings of the National Academy of Sciences 105: 7332-7338.

7. Lister R, Pelizzola M, Dowen RH, Hawkins RD, Hon G, et al. (2009) Human DNA methylomes at base resolution show widespread epigenomic differences. Nature 462: 315–322.

8. Lister R, Pelizzola M, Kida YS, Hawkins RD, Nery JR, et al. (2011) Hotspots of aberrant epigenomic reprogramming in human induced pluripotent stem cells. Nature 471: 68–73.

9. Hon GC, Hawkins RD, Caballero OL, Lo C, Lister R, et al. (2012) Global DNA hypomethylation coupled to repressive chromatin domain formation and gene silencing in breast cancer. Genome Research 22: 246–258.

10. Heyn H, Vidal E, Sayols S, Sanchez-Mut JV, Moran S, et al. (2012) Whole-genome bisulfite DNA sequencing of a DNMT3B mutant patient. Epigenetics 7: 542–550.

11. Heyn H, Li N, Ferreira HJ, Moran S, Pisano DG, et al. (2012) Distinct DNA methylomes of newborns and centenarians. Proceedings of the National Academy of Sciences 109: 10522–10527.

12. Hansen KD, Timp W, Bravo HC, Sabunciyan S, Langmead B, et al. (2011) Increased methylation variation in epigenetic domains across cancer types. Nature Genetics 43: 768–775.

13. Berman BP, Weisenberger DJ, Aman JF, Hinoue T, Ramjan Z, et al. (2012) Regions of focal DNA hypermethylation and long-range hypomethylation in colorectal cancer coincide with nuclear lamina-associated domains. Nature Genetics 44: 40–46.

14. Laurent L, Wong E, Li G, Huynh T, Tsirigos A, et al. (2010) Dynamic changes in the human methylome during differentiation. Genome Research 20: 320–331.

15. Martins-Taylor K, Schroeder DI, LaSalle JM, Xu ML, Ren-He (2012) Role of DNMT3B in the regulation of early neural and neural crest specifiers. Epigenetics 7: 71–82.

16. Li Y, Zhu J, Tian G, Li N, Li Q, et al. (2010) The DNA methylome of human peripheral blood mononuclear cells. PLoS Biol 8: e1000533.

17. Zeng J, Konopka G, Hunt B, Preuss T, Geschwind D, et al. (2012) Divergent whole-genome methylation maps of human and chimpanzee brains reveal epigenetic basis of human regulatory evolution. The American Journal of Human Genetics 91: 455–465.

18. Schroeder DI, Lott P, Korf I, LaSalle JM (2011) Large-scale methylation domains mark a functional subset of neuronally expressed genes. Genome Research 21: 1583–1591.
